# Evaluation of B_1_ inhomogeneity effect on DCE-MRI data analysis of brain tumor patients at 3T

**DOI:** 10.1186/s12967-017-1349-7

**Published:** 2017-12-02

**Authors:** Anirban Sengupta, Rakesh Kumar Gupta, Anup Singh

**Affiliations:** 1 Centre for Biomedical Engineering, IIT Delhi, New Delhi, India; 2 Department of Radiology, Fortis Memorial Research Institute, Gurgaon, India; 3 Department of Biomedical Engineering, AIIMS Delhi, New Delhi, India; 4 Centre for Biomedical Engineering, IIT Delhi, Block-II, Room No. 299, Hauz Khas, New Delhi, 110016 India

**Keywords:** DCE-MRI, B_1_ inhomogeneity, Flip-angle errors, Glioma grading, Brain-tumor

## Abstract

**Background:**

Dynamic-contrast-enhanced (DCE) MRI data acquired using gradient echo based sequences is affected by errors in flip angle (FA) due to transmit B_1_ inhomogeneity (B_1_inh). The purpose of the study was to evaluate the effect of B_1_inh on quantitative analysis of DCE-MRI data of human brain tumor patients and to evaluate the clinical significance of B_1_inh correction of perfusion parameters (PPs) on tumor grading.

**Methods:**

An MRI study was conducted on 35 glioma patients at 3T. The patients had histologically confirmed glioma with 23 high-grade (HG) and 12 low-grade (LG). Data for B_1_-mapping, T_1_-mapping and DCE-MRI were acquired. Relative B_1_ maps (B_1rel_) were generated using the saturated-double-angle method. T_1_-maps were computed using the variable flip-angle method. Post-processing was performed for conversion of signal–intensity time (S(t)) curve to concentration–time (C(t)) curve followed by tracer kinetic analysis (K^trans^, Ve, Vp, Kep) and first pass analysis (CBV, CBF) using the general tracer-kinetic model. DCE-MRI data was analyzed without and with B_1_inh correction and errors in PPs were computed. Receiver-operating-characteristic (ROC) analysis was performed on HG and LG patients. Simulations were carried out to understand the effect of B_1_ inhomogeneity on DCE-MRI data analysis in a systematic way. S(t) curves mimicking those in tumor tissue, were generated and FA errors were introduced followed by error analysis of PPs. Dependence of FA-based errors on the concentration of contrast agent and on the duration of DCE-MRI data was also studied. Simulations were also done to obtain K^trans^ of glioma patients at different B_1rel_ values and see whether grading is affected or not.

**Results:**

Current study shows that B_1rel_ value higher than nominal results in an overestimation of C(t) curves as well as derived PPs and vice versa. Moreover, at same B_1rel_ values, errors were large for larger values of C(t). Simulation results showed that grade of patients can change because of B_1_inh.

**Conclusions:**

B_1_inh in the human brain at 3T-MRI can introduce substantial errors in PPs derived from DCE-MRI data that might affect the accuracy of tumor grading, particularly for border zone cases. These errors can be mitigated using B_1_inh correction during DCE-MRI data analysis.

**Electronic supplementary material:**

The online version of this article (10.1186/s12967-017-1349-7) contains supplementary material, which is available to authorized users.

## Background

Dynamic contrast-enhanced (DCE) MRI [1] is widely used for characterization and diagnosis of intracranial mass lesions [2]. A number of studies have shown the potential of DCE-MRI in diagnosis and treatment of brain tumor [3–6]. DCE-MRI has been in clinical and pre-clinical practice for more than two decades. Various mathematical models exist for analyzing DCE-MRI data [1, 7–13]. Using the General Tracer Kinetic Model (GTKM) model [1], DCE-MRI data can be used for extraction of various parameters viz. tracer kinetic parameters like volume transfer rate (K^trans^), volume of extravascular extracellular space (Ve), plasma volume (Vp), as well as first pass analysis parameters like cerebral blood volume (CBV) and cerebral blood flow (CBF).

In general, DCE MRI data is acquired using fast Gradient Recalled Echo (GRE) sequences like SPGR/TFE. This makes the signal intensity of DCE-MRI dependent upon FA. MRI images which are acquired using gradient echo based sequences are sensitive to B_1_ inhomogeneity at high field MRI scanner like 3T [14] up to 9T [15] depending upon RF coil and type of tissue used. This field inhomogeneity introduces flip angle (FA) related errors in signal intensity. Moreover, the FA errors can propagate to further quantitative analysis.

In a previously reported study [16] on breast tissue and simulated data, propagation of FA errors in DCE-MRI data was investigated and it was reported that errors in K^trans^ and Ve vary non-linearly with FA. Propagations of FA related errors to enhancement ratio, relative change between pre and post contrast, was previously reported for breast tissue [17]. Recently, another study on the effect of transmit B_1_ inhomogeneity on tracer kinetic analysis of DCE-MRI data from breast cancer patients was also reported [18]. In this study effect of B_1_ inhomogeneity on pre-contrast T_1_ and kinetic parameters (K^trans^ and Ve) were reported. Propagations of FA-based errors on AIF estimations has also been reported for prostate tissue at 3T [19]. Thus there is a need for a systematic study using simulations and in vivo data, for evaluating propagation of errors on computed concentration and various perfusion parameters in the human brain.

Measurement of the T_1_ parameter is a pre-requisite for DCE-MRI analysis. A number of methods based upon inversion recovery [20, 21], Look locker [22], MOLLI [23], multiple Fast Spin Echo (FSE) image [24], and multiple FA [25, 26] are available for T_1_ estimation. T_1_ estimation based upon multiple FA is widely used due to shorter data acquisition time. However, T_1_ estimation using multiple FA based methods are sensitive to B_1_ field inhomogeneity [27–29]. In such cases, correction of T_1_ maps for B_1_ inhomogeneity is performed before further DCE MRI data analysis [30–34].

In the current study, in vivo DCE-MRI data and B_1_ field map data from human brain tumor patients were acquired at 3T MRI to evaluate the effect of B_1_ inhomogeneity at different stages of DCE-MRI analysis. Corrections for B_1_ inhomogeneity were applied during DCE-MRI signal to concentration conversion. In addition, T_1_ map data was also acquired using multiple flip angles and corrected for B_1_ inhomogeneity. Simulations were performed to evaluate systematically the propagation of FA errors on DCE-MRI data analysis. The clinical significance of B_1_ inhomogeneity was also investigated using statistical analysis and simulation studies.

## Methods

### Study population

The study protocol was pre-approved by the Institutional Review Board of the Institute and all subjects provided written informed consent before MR scanning. The IRB Approval Number for this study is 2013-001IP-05. Thirty-five patients (Male = 25, Female = 10) having a mean age of 50.54 ± 15.36 years (age range 16–77 years) were recruited for the study. The patients had histologically confirmed glioma with 23 high grade (HG) [21 Grade IV and 2 Grade III] and 12 low grade (LG) [11 Grade II and 1 Grade I]. Grading was done as per World Health Organization guidelines.

### MRI measurements

All MRI experiments were performed at 3T whole-body MRI system (Ingenia, Philips Healthcare, The Netherlands) using a 16 channel receive only coil. In this study, Multi Transmit parallel RF transmission was used to acquire MRI data. MRI protocol for this study included a tri-plane localizer acquiring conventional images for brain tumor patient, data for T_1_ maps, data for B_1_ maps and DCE-MRI data.

For T_1_ mapping, 3D T_1_W Turbo field echo (TFE) images were acquired with four FAs of 3°, 6°, 10° and 15°. Twelve slices, covering the tumor part, were acquired. Other MRI scan parameters were: slice thickness = 6 mm; FOV = 240 × 240 mm^2^; matrix size = 256 × 256; TR/TE of 6.0/2.1 ms.

MRI data for B_1_ map was also collected for all the subjects. Saturated dual angle method [30], with two FA interleaved approach, was used for B_1_ mapping. 2D TSE readout was used to acquire saturated dual angle images corresponding to 60° and 120°. FOV and number of slices were same as used in T_1_W TFE images. TR/TE of 600/40 ms was used to acquire images.

In the final step, DCE perfusion imaging was performed using a T_1_ Turbo field echo sequence (TR/TE = 4.45/2.01 ms; FA = 10; slice thickness = 6 mm; FOV = 240 × 240 mm^2^; matrix size = 256 × 256). At the fourth time point of the 3D-DCE-MRI data acquisition, 0.1 mmol/kg body weight of gadobenate dimeglumine Gd-BOPTA (Multihance, Bracco) was administered intravenously with the help of a power injector at a rate of 3.0 ml/s, followed by a bolus injection of a 30-ml saline flush [11]. A series of 384 images, 32 time points for 12 slices, were acquired with a temporal resolution of 3.9 s.

### Quantification of perfusion parameters

In the current study, DCE-MRI data analysis involved following steps:

#### B_1_ mapping

The B_1_ map was generated using saturated double angle based method [30]. Two images are acquired: I1 and I2 such that the tip angle of I2 is twice of I1. All other signal-affecting sequence parameters are kept constant. If the effects of T_1_ and T_2_ relaxation can be neglected, then it can be shown that

1 $$\theta = \cos^{ - 1} \left( {\frac{{I_{2} }}{{2{\cdot}I_{1} }}} \right)$$

Here $\theta$ corresponds to the tip angle that vary with the spatially varying B_1_ field. I_1_ and I_2_ corresponds to images obtained at FA 60° and 120° respectively. Computed angle $\theta$ was divided by angle of 60° degree to generate relative B_1_ (B_1rel_) map. True or nominal value corresponds to B_1rel_ = 1.

Mean (±SD) value of B_1rel_ map for all slices of 35 patients were computed and plotted. This has been done on both entire brain region covering all slices and on entire tumor region of a particular slice to estimate the B_1_ inhomogeneity range among these patients.

#### Estimation of T_1_

T_1_ map was computed using previously reported multiple FA based method [35]. The FAs used in the current study were 3°, 6°, 10° and 15°. T_1_ maps using multiple FA based method were generated by fitting the pixel-wise image intensities of the above-mentioned FAs to Eq. (2) (described in the next section) using a non-linear least-square fitting routine in MATLAB. The ‘lsqcurvefit’ routine in MATLAB with lower and upper bound of 200 and 5000 ms and an initial guess of 1000 ms was used for curve fitting. The lsqcurvefit routine uses the trust-region-reflective algorithm. For obtaining B_1_ inhomogeneity corrected T_1_ map, all FAs were corrected pixel-wise for B_1_ inhomogeneity using B_1rel_ map [30].

#### Effect of B_1_ inhomogeneity on DCE-MRI data and its corrections

Signal intensity for SPGR/TFE signal is represented by the following equation:

2 $$S = \frac{{M_{0} .\sin \left( \theta \right).e^{{ - \frac{TE}{{T_{2}^{*} }}}} .\left( {1 - e^{{ - \frac{TR}{{T_{1} }}}} } \right)}}{{1 - \cos \left( \theta \right).e^{{ - \frac{TR}{{T_{1} }}}} }}$$

where $M_{0}$ is the equilibrium longitudinal magnetization. $M_{0}$ is given as $G$. $\rho$ where $G$ is the gain and $\rho$ is the proton density. TR and TE represents repetition and echo times, T_1_ and T_2_^*^ are relaxation times and $\theta$ is the FA.

The signal S is a function of θ. Therefore, DCE MRI data which is acquired before, during and after intravenous injection of contrast agent show dependence on FA. B_1_ field inhomogeneity results in variation of nominal FA and hence in the signal intensity time curve (S(t)) of DCE-MRI data. These variations propagate to further DCE-MRI analysis. Due to B_1_ inhomogeneity, voxelwise FA (at *i*th voxel) is given by the following equation:

3 $$\theta (i) = \theta_{nominal} .B_{1rel} (i)$$

Here $\theta (i)$ is the modified FA at *i*th voxel, according to B_1rel_ value at corresponding voxel, and $\theta_{nominal}$ is the nominal value specified by user in MRI protocol. Since, data provided by scanner at a voxel (*i*) correspond to *θ*(*i*) given by Eq. (3) therefore, during quantitative analysis, *θ*(*i*) should be used instead of $\theta_{nominal}$ in order to remove the effect of B_1_ field inhomogeneity.

Since we are mainly interested in quantitative analysis of DCE-MRI data and the first step is the conversion of S(t) toC(t), therefore, it’s important to correct C(t) for B_1_ inhomogeneity. In this study, S(t) was converted to C(t) using previously described procedure [24].

Contrast agent changes the relaxation rates of tissues. The increase in relaxation rates are linearly related to contrast concentration in the tissue:

4 $$\frac{1}{{T_{1}^{ * } }} = \frac{1}{{T_{10} }} + r1{\cdot}C(t) \quad {\text{and}} \quad \frac{1}{{T_{2}^{ * } }} = \frac{1}{{T_{20} }} + r2{\cdot}C(t)$$

Here r1 = 5.9 l/mmol/s and r2 = 17.5 l/mmol/s at 37 °C and 3T [36]. After injection of contrast, the signal from an SPGR sequence is given by [24]:

5 $$S(t) = G \cdot \rho \cdot \exp \,( - TE \cdot (T_{20}^{ - 1} + r2 \cdot C(t))) \cdot \sin (\theta ) \cdot \frac{{1 - \exp \,( - TR\,(T_{10}^{ - 1} + r1 \cdot C(t)))}}{{1 - \cos (\theta ) \cdot \exp \,( - TR\,(T_{10}^{ - 1} + r1 \cdot C(t)))}}$$

For this particular study, TE is small enough to neglect its effect. Briefly, the above equation can be reduced to

6 $$\frac{S(t)}{S(0)} = \frac{{K_{0} \cdot E2 \cdot (1 - E3)}}{1 - \cos (\theta ) \cdot E3}$$

where $${{K_{0} = (1 - \cos (\theta ) \cdot e^{{( - TR/T_{10} )}} )} \mathord{\left/ {\vphantom {{K_{0} = (1 - \cos (\theta ) \cdot e^{{( - TR/T_{10} )}} )} {(1 - e^{{( - TR/T_{10} )}} )}}} \right. \kern-0pt} {(1 - e^{{( - TR/T_{10} )}} )}}$$, $$E3 = e^{{\left( { - TR.\left( {\frac{1}{{T_{10} }} + r1 \cdot C(t)} \right)} \right)}}$$.

T_10_ is the value of T_1_ of tissues before injection of contrast agent. C(t) is the concentration of Gd-BOPTA at time ‘t’ in the tissue and C(0) = 0. Since in this study, T_10_ is estimated separately, therefore, Eq. (6) is a non-linear equation with only 1 unknown parameter C(t). This equation was solved to obtain the value of C(t) at different time points.

While converting S(t) to C(t), voxelwise FA as given by Eq. (3) was used. Here only pre-contrast T_1_ values (T_10_) are required as opposed to T_1_(t) at each time point during direct correction of S(t). This step resulted in B_1_ inhomogeneity corrected C(t) curves. For generating C(t) without B_1_ inhomogeneity correction, we used $\theta_{\text{nominal}}$ as the $\theta$ in Eq. (6).

#### Estimation of AIF

In this study, we have used automatically detected local arterial input function (AIF) for each glioma patient using previously described procedure [10]. Briefly, the method for automatic extraction of AIF is based on the features exhibited by the concentration–time curve in vascular voxels. The main features of concentration–time curve at arterial voxels are: early arrival of contrast or early bolus arrival time, high peak value (during first pass), early arrival of time to peak; sharp uptake (high gradient value) of contrast agent, and high average value of concentration of contrast.

#### Tracer kinetic model fitting

In the current study we have used generalized tracer kinetic model (GTKM) described previously [1] for estimating kinetic parameters particularly volume transfer rate (K^trans^), volume of extracellular extravascular space (Ve), plasma volume (Vp) and Kep which is the ratio of K^trans^ and Ve. Briefly, following equation of GTKM is used:

7 $$C_{T} (t) = V_{p} C_{p} \left( t \right) + K^{trans} \mathop \int \limits_{0}^{t} C_{p} (\tau )e^{Kep(\tau - t)} d\tau$$

where C_T_ is the concentration of contrast in tissue/voxel and Cp is AIF. Voxel-wise fitting of GTKM was performed using in-house written programs in MATLAB and inbuilt MATLAB routine function ‘lsqcurvefit’ which uses the trust-region-reflective algorithm. The upper bound and lower bound values for K^trans^, Kep and Vp was [1, 2, 8] and [0, 0, 0]. Ve was estimated as the ratio of K^trans^ and Kep. Tracer kinetic model fitting followed the previously described procedure [24].

#### First pass analysis

First pass analysis [37] for estimation of hemodynamic maps of CBF, CBV and corrected CBV (CBV_Corr) were carried out using previously described procedure [24]. It needs to be mentioned here that there is an overestimation of CBV in the regions where the contrast leaks into extravascular extracellular space because in this case, it represents the volume of contrast in intravascular as well as in leakage space. CBV_Corr represents the volume of blood only in the intravascular space and is to be estimated by removing the contribution of fractional leakage space volume (Ve) from the CBV.

### Sample size estimation

To evaluate the clinical significance of B_1_ inhomogeneity statistical analysis was performed. The sensitivity and specificity of the perfusion parameters were calculated using receiver-operating characteristic (ROC) analysis. To estimate the sample size required for differentiation between HG and LG patients, it was assumed that the area under the ROC curve (AUC) of 0.8 is significant from the null hypothesis value 0.5 (meaning no discriminating power). For 95% power and a 0.1 level of significance, the minimum sample size required for positive (HG) and negative responses (LG) was 22 and 11 respectively. A total of 23 high grade (HG) which is Grade-4 and Grade-3 combined and 12 low grade (LG) which is Grade-2 and Grade-1 combined, patients were taken for this study.

### Statistical analysis

Within the tumor region of a particular slice, 2–3 circular regions of 5 voxel radius showing high values of post B_1_ corrected CBV_Corr were manually placed and the one showing maximum value was chosen as Region of Interest (ROI). All perfusion parameters before B_1_ correction and after B_1_ correction were obtained from the ROIs. Relative percentage error (RPE) for with and without B_1_ inhomogeneity correction, were computed for C(t) and perfusion parameters corresponding to these ROIs. RPE was defined as:

$${\text{RPE}} = \frac{{\left( {{\text{Observation before correction}} - {\text{ observation after correction}}} \right)}}{\text{Observation after correction}} \times 100$$

These perfusion parameters were further used for ROC analysis. Cutoff value at which the average of sensitivity and specificity is maximized and area under the curve of ROC analysis were obtained for grading between HG and LG gliomas. Histologically confirmed grades were taken as a gold standard. All analysis was done using SPSS statistics software package version 16.0. A paired t test with two-tailed distribution was done to find out whether the change in each perfusion parameter before and after B_1_ correction is significant or not. The Bland–Altman (BA) plot was also computed to compare values of perfusion parameters before and after B_1_ correction. In this graphical method, the differences between the two techniques are plotted against the averages of the two techniques. Here the difference of parameters is plotted against post-B_1_ correction technique since that is the reference method. The mean difference should be less than the limits of agreement which is defined as the 95% CI of the mean difference, for the agreement between results of with and without correction.

### Simulations

Simulations were carried out to understand the effect of B_1_ inhomogeneity on DCE-MRI data analysis in a systematic way. Equation (6) was used for simulating S(t) curves mimicking those in tumor tissues. Parameters used in the simulation are shown in Table 1. For simulated AIF we have used parameters as reported in the literature [38]. Three cases were simulated in this study. In the 1st case, C(t) values from 0:0.01:0.5 mmol/l were used to generate S(t) curve using Eq. (6). Now while converting back from S(t) to C(t), errors in FA were introduced for mimicking B_1_ field inhomogeneity effect. This was done to understand the effect of initial concentration amount on an error in C(t) due to different B_1_ inhomogeneity errors. In the 2nd case, C(t) curves mimicking response of tumor tissue were generated without and with different B_1_ inhomogeneity errors. This was done to evaluate the error in C(t) due to B_1_ inhomogeneity. In 3rd case, three C(t) curves having different concentration amounts (mimicking those in tumor tissue) were generated and effect of B_1_ inhomogeneity errors in various parameters were computed. This involved using different K^trans^ values [0.1, 0.2, 0.3] as the starting values to generate three different C(t) while keeping Kep and Vp values fixed as shown in Table 1. The goal of this study was to estimate the change in perfusion parameters as a result of change in C(t) curves due to FA error. It needs to be mentioned that in simulation studies the FA for dynamic study is 10° which is same as the FA used in in vivo data.

**Table 1:** Parameters used in simulations

Parameters	Values
T_10_	1500 ms
TR	4.45 ms
Flip angle (FA)	10
r1	5.9 l/mmol/s
r2	17.5 l/mmol/s
Scaling factor	1000
Time points	32
B1 inhomogeneity	0.8–1.2 at step size of 0.05
[K_ep_, V_p_] at staring point	[0.5, 0.02]

Another simulation study was done to illustrate the clinical significance of B_1_ inhomogeneity correction. 5 HG (Grade3 and Grade-4) and 5 LG (Grade-1 and Grade-2) patients were randomly selected. K^trans^ values after B_1_ correction corresponding to the chosen ROI were used. B_1rel_ was altered from a range of 0.75–1.25 to see how it influences the perfusion parameters. The C(t) values were kept same for all of them to study the influence of B_1_ exclusively on perfusion parameters. The threshold for grading used in this study was same as that obtained from ROC analysis after B_1_ correction.

## Results

**Fig. 1:**
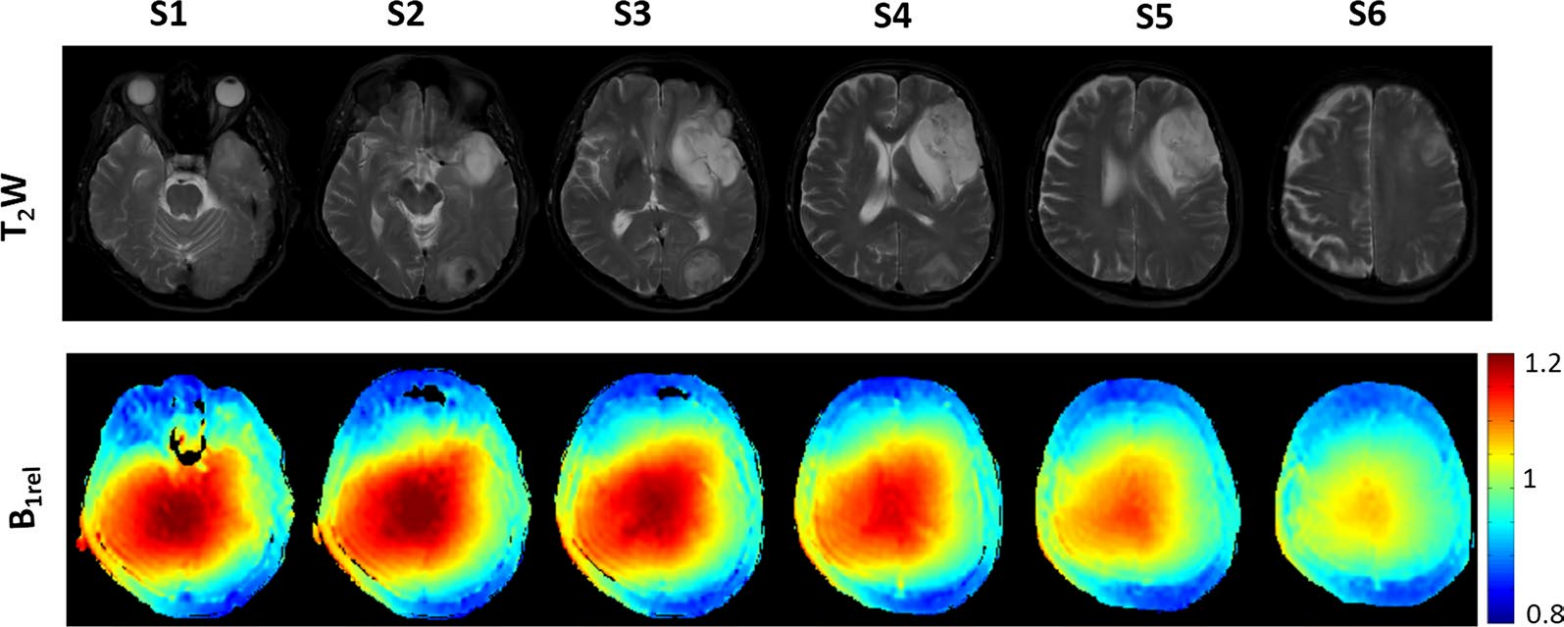
Shows T_2_ W images (1st row) and B_1rel_ maps (2nd row) from six alternate slices of a human brain tumor patient. Voxels with B_1rel_ value of 1 corresponds to the nominal B_1rel_ value

In the current study, it was evident that B_1_ inhomogeneity was present across different MRI image slices of human brain. Figure 1 shows B_1rel_ maps of multiple brain slices of one subject. B_1rel_ values were higher than the nominal value of 1 in the central part of the brain. In the peripheral region of the brain, B_1rel_ values are found to be less than the nominal value of 1.

**Fig. 2:**
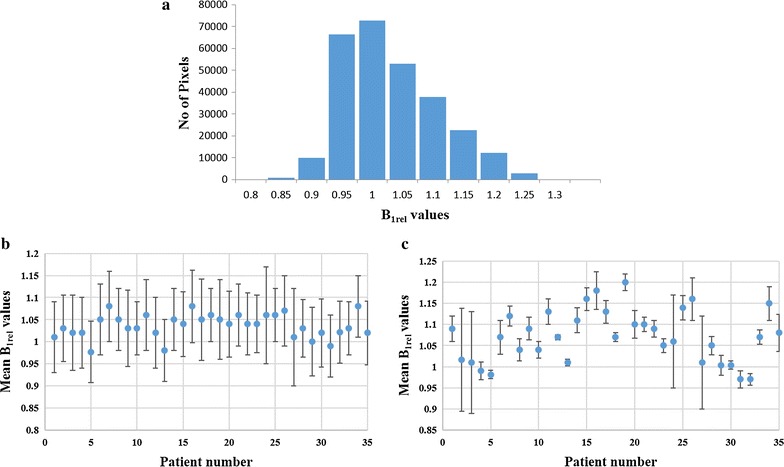
It shows the B_1_ distribution obtained from B_1rel_ maps. **a** The histogram plot of B_1rel_ values on all slices of Brain of a particular patient. **b** The distribution (mean ± SD) of B_1rel_ map values in all slices of the brain of 35 subjects and **c** the same in tumor region of 35 subjects

Histogram plot of B_1rel_ values for 12 brain slices of a particular patient in Fig. 2a shows a broad distribution of B_1rel_ values which extends to both sides of the nominal value. For this subject, B_1rel_ values range in the brain slices were [0.85–1.25] with the maximum value found between 1 and 1.05. Figure 2b, c show plots of B_1rel_ values (mean ± SD) of the entire brain and tumor tissue respectively for 35 different subjects. Large variations in B_1rel_ values of entire brain was observed in each of 35 patients. More importantly, it was observed that mean B_1rel_ values ranged from 3% below nominal to 20% above nominal in the tumor region among different patients.

Table 2 shows B_1rel_ values and corresponding relative percentage error (average value ± S.D) of different parameters at the previously mentioned ROI in tumor region for 35 different patients. B_1rel_ values ranging from 0.95 to 1.25 at an interval of 0.05 has been shown. RPE of all perfusion parameters shows an increasing trend with an increase of B_1rel_ values. For B_1rel_ values less than the nominal range (which is equal to 1), RPE of perfusion parameters is negative and vice versa. Kep’s values are not presented in the table as it is not an independent parameter, being the ratio of K^trans^ and Ve.

**Table 2:** Relative percentage error (RPE) in parameters, derived from DCE-MRI data analysis

	Relative % error (mean ± S.D)					
B1	K^trans^	Ve	Vp	CBV	CBF	CBV_Corr
0.95–1	− 0.85 ± 0.70	− 0.96 ± 0.87	− 0.42 ± 1.33	− 1.02 ± 0.794	− 1.22 ± 0.786	− 0.84 ± 0.815
1–1.05	0.26 ± 1.80	9.10 ± 17.99	1.25 ± 0.80	1.28 ± 0.50	1.17 ± 0.408	1.66 ± 1.91
1.05–1.10	3.77 ± 1.19	5.50 ± 2.80	3.03 ± 1.65	3.49 ± 0.95	3.27 ± 1.47	2.75 ± 0.76
1.10–1.15	5.62 ± 2.52	19.12 ± 13.10	6.64 ± 3.41	6.01 ± 1.79	6.44 ± 2.48	4.19 ± 1.23
1.15–1.20	9.74 ± 2.44	14.78 ± 9.84	7.22 ± 1.70	8.48 ± 0.92	9.49 ± 3.63	7.12 ± 1.28
1.20–1.25*	12.91	12.86	9.70	10.51	10.52	9.69

RPE of each parameter from brain tumor of 35 patients were grouped based upon range of B1_rel_ value

* This group had only one patient and hence no S.D

**Fig. 3:**
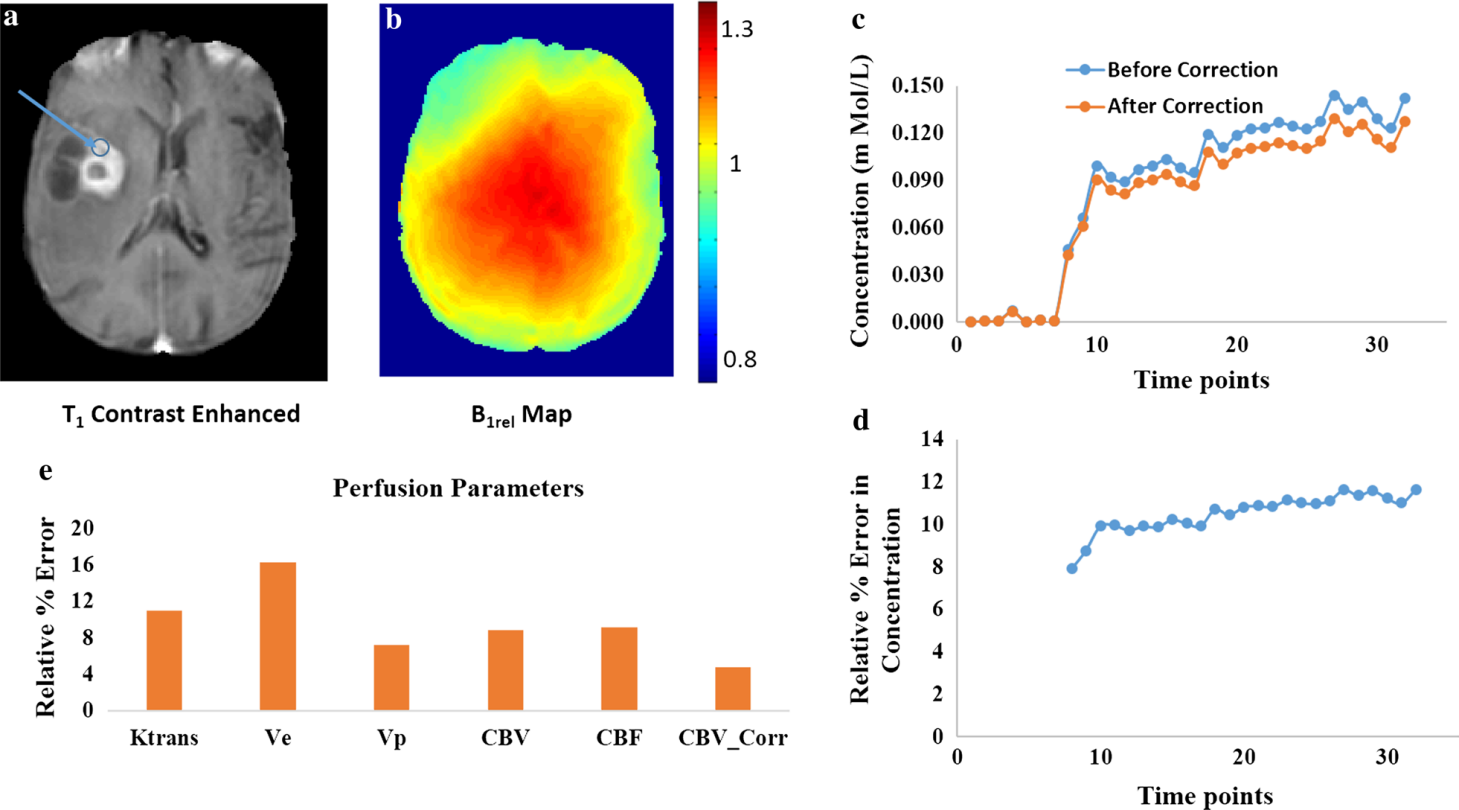
It shows data from a subject with brain tumor having B_1rel_ value 1.15 in the tumor region pointed by the arrow. **a** The post contrast T_1_W image. **b** The relative B_1_ map (B_1rel_) of the corresponding slice. **c** The concentration curve over 32 time points at the tumor location denoted by the arrow in (**a**) before and after B_1_ inhomogeneity correction. **d** The relative error in concentration over the time points. The first 7 time points are excluded as contrast reaches tissue at ~ 8th time point. **e** The percentage error in perfusion parameters before and after B_1_ correction

Figure 3 show data from a patient with glioblastoma (GBM) having a B_1rel_ value of 1.15 in an ROI in the tumor region pointed by the arrow. An enhancing ring like tumor region is visible in that slice. In the ROI a steep rise in concentration was observed because of contrast leakage into the tumor due to the Blood Brain Barrier breakdown. In Fig. 3b B_1rel_ map shows the inhomogeneous B_1_ distribution in the particular slice of brain. Figure 3c shows that C(t) at the tumor location reduces after B_1_ inhomogeneity correction over 32 time points. Figure 3d shows that there is a relative percentage error of 11.65% in concentration at the end of 32 time points. Bolus arrival time for this ROI was at the 8th time point. Therefore, first 7 time points were excluded from error computation. Figure 3e shows the relative percentage error in various perfusion parameters due to B_1_ inhomogeneity. It shows that a B_1rel_ value greater than nominal value (before correction) results in an overestimation of parameters CBV, CBV-Corr, CBF, K^trans^, Ve, Vp. For this subject, Ve showed maximum RPE of ~ 16%.

**Fig. 4:**
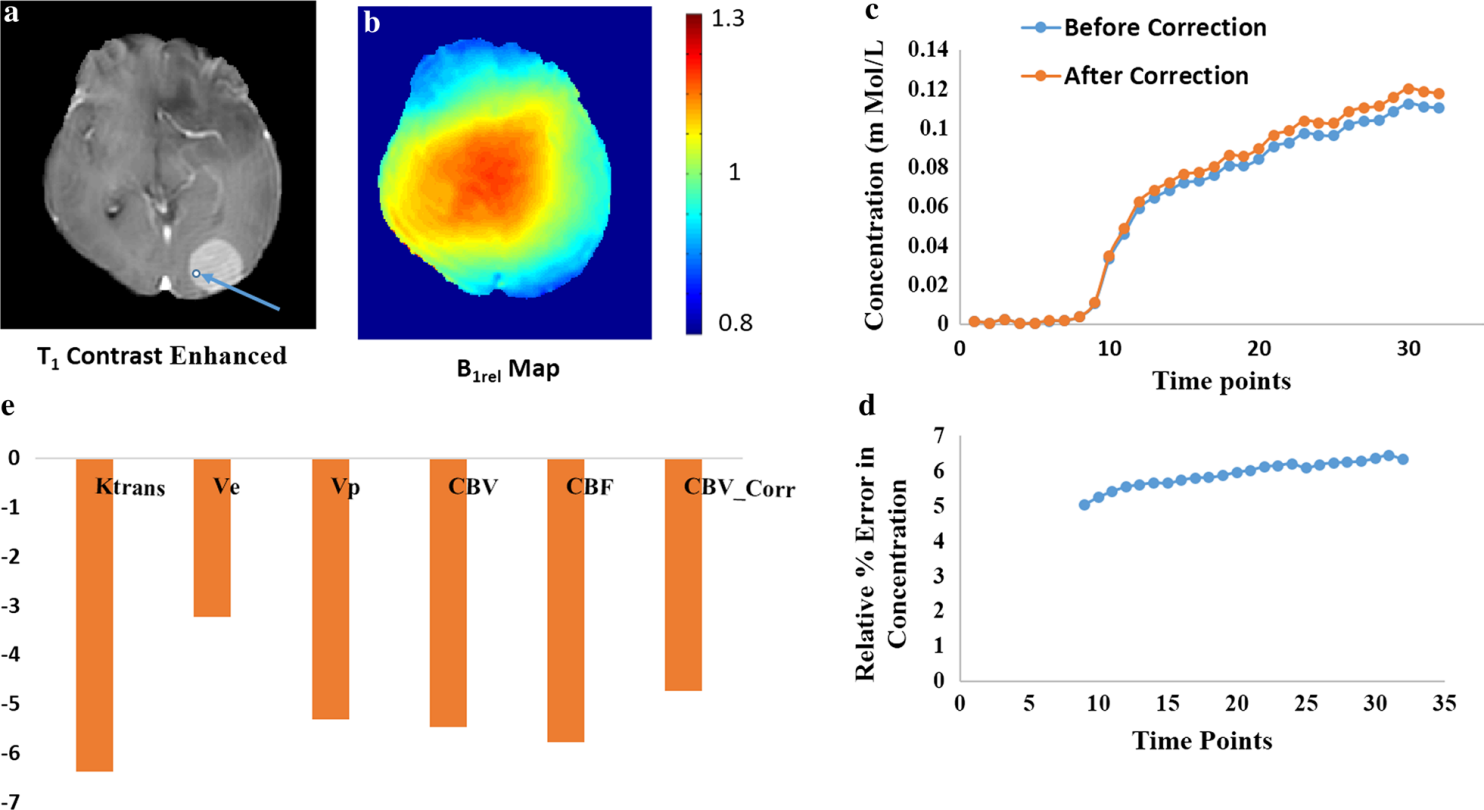
It shows data from a subject with brain tumor having a B_1rel_ value of 0.94 in the tumor region pointed by the arrow. **a** The post contrast T_1_W image. **b** The relative B_1_ map (B_1rel_) of the corresponding slice. **c** The concentration curve over 32 time points at the tumor location denoted by the arrow in (**a**) before and after B_1_ inhomogeneity correction. **d** The relative error in concentration over the time points. The first 8 time points are excluded as contrast reaches tissue at ~ 9th time point. **e** The percentage error in perfusion parameters before and after B_1_ correction

Figure 4 shows data from a subject with brain tumor having a B_1rel_ value of 0.94 (lower than nominal value) in the tumor region. Figure 4a shows the tumor location in the post-contrast T_1_W image. Figure 4b shows that the B_1rel_ value in the ROI taken from tumor region is below the nominal value. Figure 4c shows the C(t) at the tumor ROI is increased after B_1_ Correction. Figure 4d shows that there is a relative percentage error of 6.3% in concentration at the end of 32 time points. Bolus arrival time for this ROI was around 9th time point. Therefore, first 8 time points were excluded from error computation. The lower value of B_1rel_ results in an underestimation of C(t), which was corrected after B_1_ inhomogeneity correction. Similarly, all perfusion parameters, show underestimation with a relative percentage error of K^trans^ reaching − 6.3% shown by in Fig. 4e.

Table 3 gives relative % change in variation (calculated as square of SD) of each perfusion parameter for both HG and LG patients. Mostly, it was seen that variation of perfusion parameters reduced after correction within each grade.

**Table 3:** Relative % change in variation of perfusion parameters within high and low grade patients

Parameters	High grade	Low grade
K^trans^	6.605796	15.10635
Ve	6.638113	15.83864
Vp	15.21406	1.00593
CBV	6.233672	7.027053
CBF	4.495963	− 0.80946
CBV_Corr	9.547645	6.775655

Relative % change = 100 × (variation before correction − variation after correction)/variation after correction

**Fig. 5:**
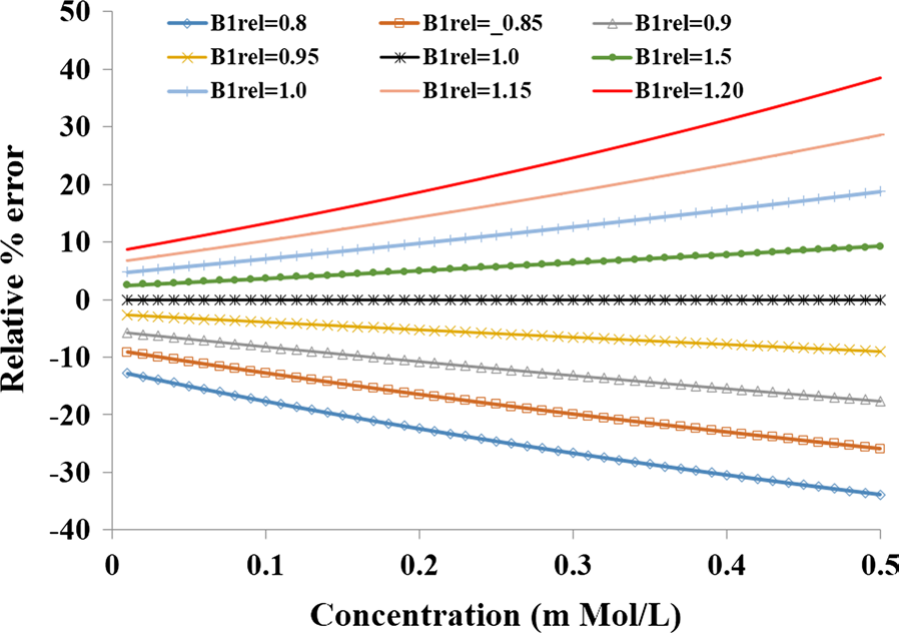
Simulation results. Plots show error in computed concentration due to an error in B_1rel_ or FA. At a particular B_1rel_, error increased with increase in concentration

**Fig. 6:**
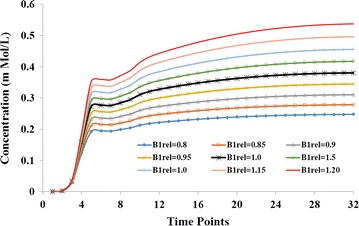
Simulation results shows errors in concentration–time curve (mimicking enhancing tumor), as a function of B_1rel_. Concentration values changed in accordance with B_1rel_. Unit time point = 3.9 s

Dependence of FA related errors, using simulations, in computed concentration on the amount of nominal concentration is shown in Fig. 5. It was observed that FA related errors increase with an increase in the amount of concentration. Figure 6 show propagation of B_1rel_ errors to simulated C(t). Simulated C(t) curve mimics those of contrast enhancing tumor region. Simulations show that values of B_1rel_ lower than nominal value results in an underestimation of C(t) while higher values result in overestimation. This behavior was similar to experimental results obtained from brain tumor patients.

**Fig. 7:**
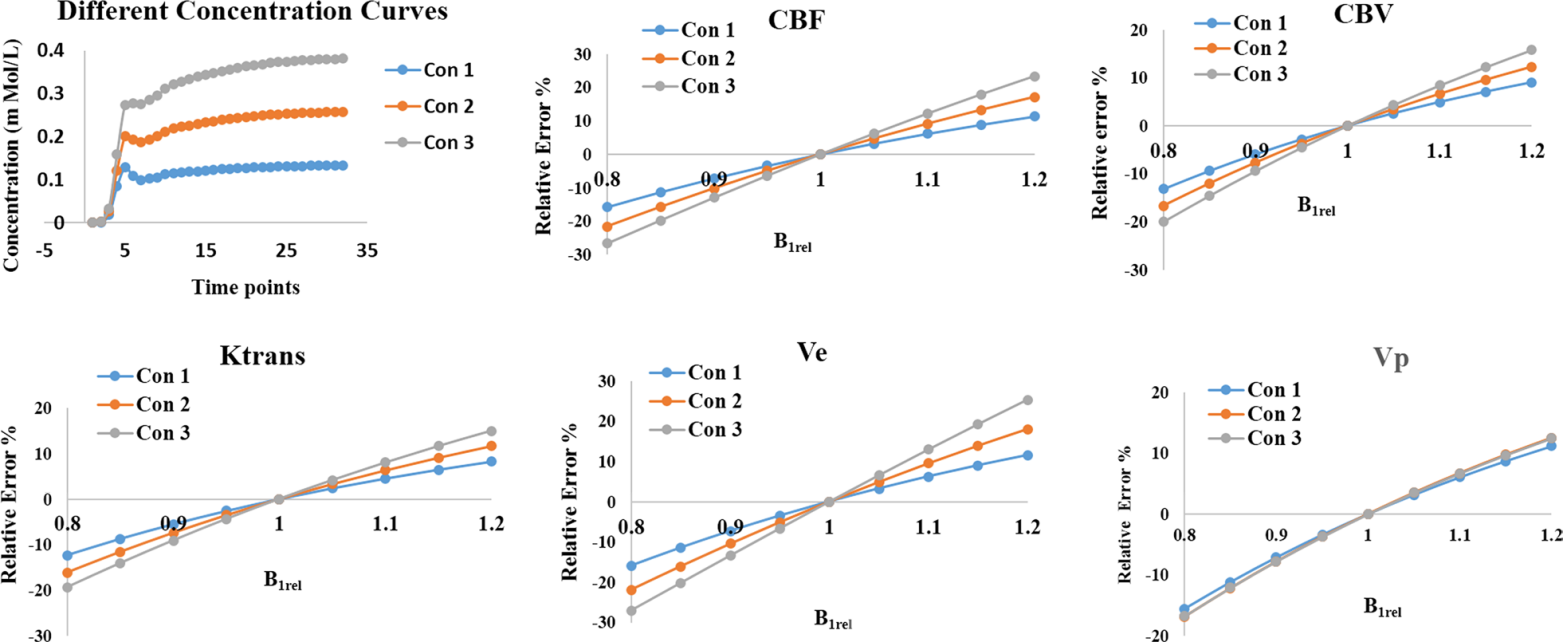
Simulation results show three different types of concentration curves and propagation of B_1_ inhomogeneity errors to various parameters. Relative percentage error of various parameters was plotted against B_1rel_ value

Simulation results shown in Fig. 7 demonstrate the dependence of B_1_ inhomogeneity related errors in perfusion parameters on the concentration of contrast agent. Overall, errors in all the parameters increased with increase in the concentration of contrast agent. Parameter Ve showed the maximum error.

**Fig. 8:**
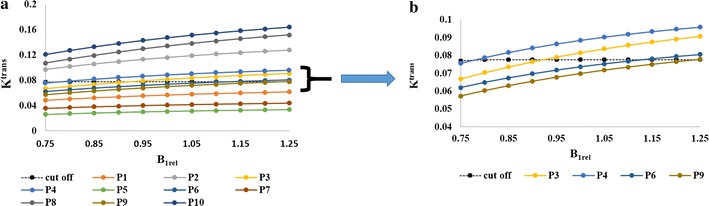
Simulation results. Scatter plot **a** demonstrates the relationship between errors introduced in B_1rel_ field and that in the kinetic parameter K^trans^ for randomly selected 5 HG and 5 LG patients. Scatter plot **b** the same figure zoomed in at the cut off region

Figure 8a shows that the cut-off for K^trans^ between HG and LG patients is 0.77. Patient 2, 8, 10 were HG and remained HG at different B_1rel_ values. Patient 1, 5, 7 were LG and remained LG with a change in B_1rel_ values. Patient 4 changed from HG to LG at B_1rel_ less than 8, patient 3 changed from HG to LG at a B_1_ value less than 0.95, patient 6 changed from LG to HG at B_1rel_ greater than 1.15 whereas patient 9′s grade changed from LG to HG at the B_1rel_ value of 1.25 or more. Figure 8b shows the same graph for borderline patients 3, 4, 6 and 9 for better visualization.

**Fig. 9:**
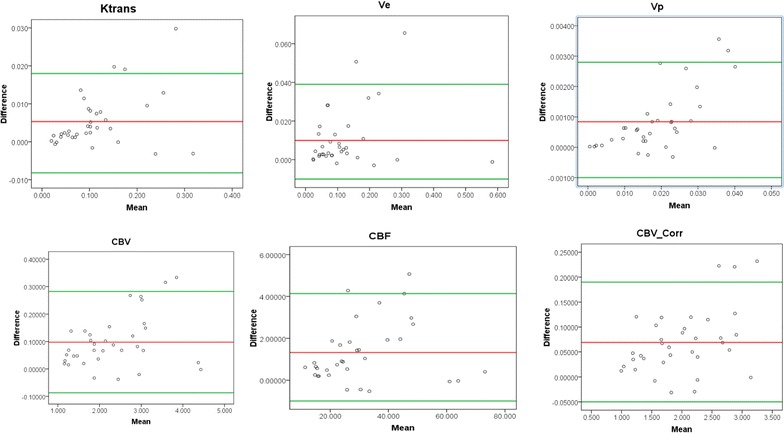
Bland–Altman plots showing variability in DCE-MRI derived perfusion parameters before B_1_ correction and after B_1_ correction

Paired t test result showed that changes in each perfusion parameter before and after correction is significantly different (p < 0.001). In Fig. 9 Bland–Altman plots for perfusion parameters showed that for each parameter the mean difference fell within the limits of agreement for the majority of subjects. However, for all metrics, two to three subjects fell outside the limits of CI.

## Discussion

In the current study, B_1_ inhomogeneity in the human brain with tumor at 3T MRI was estimated and its effect on concentration–time curves and derived perfusion parameters was evaluated using experimental as well as simulated data. Mean B_1rel_ ranged from 3% below nominal to 20% above nominal in tumor tissues of different patients, which resulted in erroneous estimates of concentrations as well as perfusion parameters. It was also observed that B_1rel_ in tumor ROI is lower than nominal for few patients whereas it was higher than nominal for the majority of patients. This resulted from the heterogeneous B_1rel_ field across the brain. Centre of the brain shows B_1rel_ values much higher than nominal compared to the periphery. Thus tumor near the central part of the brain had higher B_1rel_ values compared to a tumor in the periphery region. Simulation results provided a systematic evaluation of propagation of FA related errors in DCE-MRI data analysis. The current study shows that B_1rel_ value greater than nominal results in overestimation of C(t) curves as well as derived perfusion parameters. Similarly, B_1rel_ values lower than nominal result in underestimation of parameters. Kep being a ratio of K^trans^ and Ve didn’t provide any unique information and hence was not mentioned in the results. Preliminary results of this study have been reported [39].

One of the observations in the current study was that errors in C(t) due to B_1_ inhomogeneity increases non linearly with increase in concentration amount. The amount of concentration in contrast enhancing tumor region is different for different patients. The errors in perfusion parameters have a complex dependence on the amount of concentration and B_1rel_ value at a particular ROI.

In the current study, a 2D TSE readout technique was used to acquire B_1_ map data. This technique is intrinsical to slice selection because of slice selective refocusing pulses. So, a non-selective excitation pulse (at 2 different angles) can be used without having to go for a 3D volume acquisition. Thus the used sequence provides fast and accurate results without taking into account the slice profile of excitation pulse. Moreover, during dynamic image acquisition, an echo time of 2.1 ms was used. It was seen through some preliminary studies that changes in T_20_ at such a small TE didn’t affect the calculation of DCE parameters. Thus in this study, we have neglected the effect of T_20_.

In the current study, lower and upper bound values during T_1_ map estimation and Tracer kinetic model fitting using Matlab routines were decided based on physiological constraints of a particular tissue.

The first step in DCE MRI analysis requires pre-contrast T_10_ map calculation. In the current study, T_1_ estimation was performed using multi-flip angles. The T1 map obtained was found to be effected by B_1_ inhomogeneity. Hence, T_1_ map was also corrected for B_1_ field inhomogeneity. In the current study, relative percentage error (RPE) of DCE parameters were positive for B_1rel_ values greater than 1 and negative for B_1rel_ values less than 1. This trend is opposite to the findings of Bedair et al. [18]. This is because, in the current study, RPE is calculated with respect to results of post B_1_ correction, which is the corrected value.

There is a monotonic increase in RPE of all DCE parameters although not linearly. This is because of the different concentration amount reaching the tumor in each patient and it has been shown in simulation studies that amount of concentration reaching the tumor also plays a role in deciding RPE of perfusion parameters.

It should be mentioned here that 3D acquisition of DCE MRI data can result in inhomogeneous slab selection. However in the current study, tumor from central slice has been chosen in which inhomogeneous slab selection is not a problem.

The simulation studies have been designed to evaluate FA related error propagations in a systematic way and to verify the experimental results. Simulation studies helped in covering a wide range of concentration and B_1_ inhomogeneity, which was difficult to cover using experimental data of the current study. Moreover, experimental data results can be affected by noise, which might influence the true behavior of error propagation. Without simulations, it’s difficult to demonstrate that B_1_ inhomogeneity errors depend both on B_1rel_ value and initial contrast agent concentration. In the simulation study a T_1_ = 1500 ms, which is similar to that of enhancing tumor tissue was used to obtain the results. It was found that similar results were obtained for different T_1_ values such as 800, 1200 and 2000 ms (results not shown). In the current study, local AIF obtained from each patient data was used for DCE-MRI analysis. However, for simulation studies, global AIF was used. Global AIF can be obtained as an average of local AIFs from different patient data or based on published literature.

It can also be intuitively seen that cut off values for differentiating between grades will be always dependent on tumor location and B_1rel_ value at that ROI. Since, this will be changing from patient to patient, cut off values will also be varying arbitrarily as more and more patients are added to the study. This problem won’t arise if B_1_ correction is conducted beforehand. Thus the clinical significance of using B_1_ correction is intuitionally evident.

In a limited in vivo data set, it is not always possible to get appropriate cases where B_1_ correction can come into clinical importance. Appropriate cases are those where high B_1_ inhomogeneity coincides with ROI (obtained from the maximum CBV_Corr region) within the tumor of glioma patients. This may not happen in many glioma patients and can purely depend on chance depending on tumor ROI location and B_1_ inhomogeneity of that ROI. Thus Bland–Altman plots showed that the mean difference between before and after B_1_ corrected perfusion parameters were outside the limits of agreement for few subjects. However, a number of cases were border zone cases. In the current study, the sensitivity, specificity, and AUC of perfusion parameters from ROC analysis did not show much change between pre and post B_1_ correction to come to any conclusive decisions (results shown in Additional file 1). Hence, simulations were performed using in vivo data to demonstrate the effect of B_1_ inhomogeneity correction on the accuracy of grading. K^trans^ was chosen for simulation studies as it had maximum AUC as was found from ROC analysis (Additional file 1). It needs to be mentioned here that cutoff value of K^trans^ used in simulation studies was from B_1_ corrected results. Change in grade has been observed in those cases where the deviation of the value of the grading parameters from cutoff value is less. This suggests that B_1_ inhomogeneity may influence glioma grading in cases where perfusion parameter values are on the borderline of cut off value for separating high-grade from low-grade glioma.

It was also observed from Table 3 that for both HG and LG patients, intragroup variation of perfusion parameters reduced after B_1_ correction even with a small sample size. This observation highlights the importance of B_1_ correction when perfusion parameters are used for clinical diagnosis such as grading.

In this study, the FA used for obtaining DCE-MRI data is 10 degree, which is around two times compared to Ernst angle corresponding to TR of 4.45 ms. The nature of error propagated to DCE-MRI data can also vary depending on the FA used. For a fixed TR, B_1_ related errors on dynamic data analysis reduce with increase in FA compared to Ernst angle (observation based on simulation, result not shown). However, this also results in a reduction of SNR in DCE-MRI data. Therefore, a tradeoff is usually carried out during protocol designing to select appropriate FA for a fixed TR. On the other hand, B_1_ inhomogeneity correction enables to use DCE-MRI data corresponding to FA close to Ernst angle and hence obtain an improved SNR.

One of the limitation is the unavailability of enough patient data so as to illustrate the clinical significance of this study. However, this limitation was addressed by using simulation using in vivo study results. In this study, ROI selection was done on the basis of maximum CBV_Corr values in the tumor region. However, for accurate selection of ROI, those regions should be avoided where high CBV_Corr values coincide with blood vessels. In the current study, we have used SDA based approach for B_1_ mapping. There are many alternative sequences which can be used for B_1_ mapping. A detailed study needs to be done to investigate how the FA related errors in the quantitative analysis of DCE-MRI data varies with different B_1_ mapping approaches. Another future work in this study is to optimize the FA used for obtaining DCE-MRI data based on B_1_ inhomogeneity propagated error.

B_1_ field inhomogeneity depends upon MRI scanner field strength, type of coil as well as the type of tissue being studied. A recently reported study on breast DCE-MRI showed an average of 37% FA difference between the right and left breast [18]. B_1_ field inhomogeneity increases with increase in MR scanner field strength. For example at 7T, reported studies have shown B_1_ field inhomogeneity of ~ 50% in human brain data [33]. Such a large B_1_ field inhomogeneity can result in proportional variations in FA, which can lead to errors in DCE-MRI data analysis at 7T. In the current study we have demonstrated results for brain data at 3T; however, similar results should be observed for DCE-MRI studies of different organs as well as at ultra-high field scanner 7T.

## Conclusions

In conclusion, a substantial transmit B_1_ field inhomogeneity was observed in tumor tissues of the human brain at 3T MRI scanner. It was demonstrated that it can introduce errors in the quantitative parameters derived from DCE-MRI data, which can affect diagnosis and prognosis of patients. B_1_ inhomogeneity related errors in the DCE-MRI analysis showed dependence on B_1rel_ values, contrast agent concentration as well as on the length of DCE-MRI data. Overall, B_1_ inhomogeneity results in erroneous estimates of quantitative parameters. Correction of FA errors during conversion of S(t) to C(t) can mitigate these errors and provide an improved diagnosis.

## Additional file

**Additional file 1.** ROC analysis of different perfusion parameters before and after B_1_ correction.

## Abbreviations

B_1rel_: relative B1
S(t): signal time curve
C(t): concentration time curve
FA: flip angle
RPE: relative percentage error
HG: high grade
LG: low grade
