# Supplementary figures and images for: Evaluation of B1 inhomogeneity effect on DCE-MRI data analysis of brain tumor patients at 3T
Source: J Transl Med. 2017 Dec 2;15:242. doi: 10.1186/s12967-017-1349-7 (PMC5712076; doi:10.1186/s12967-017-1349-7)

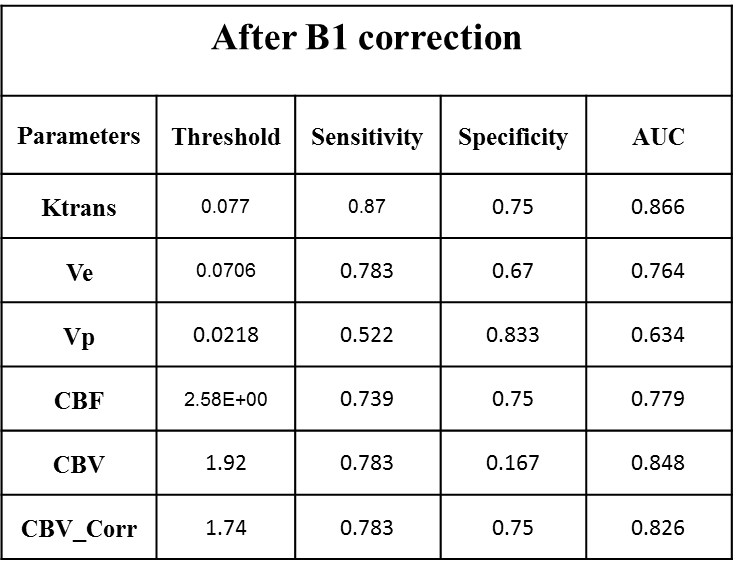

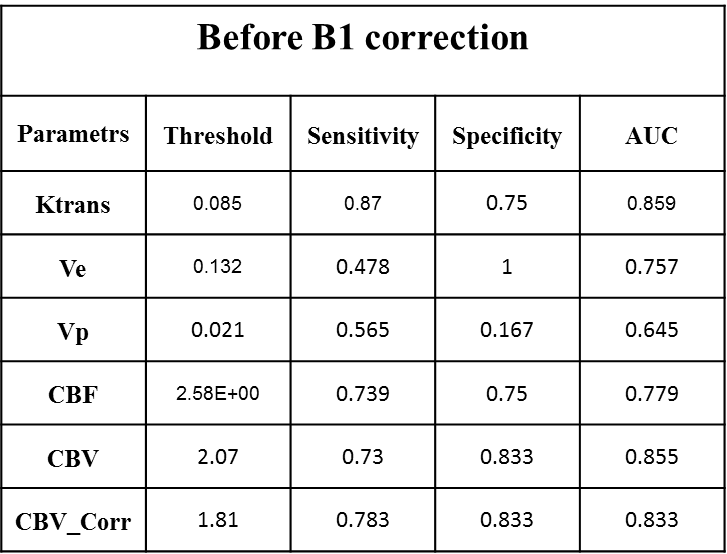


**Table S1: ROC Analysis of different Perfusion Parameters**

Supplement: Supplementary file 1 — Additional file 1. ROC analysis of different perfusion parameters before and after B1 correction. [file 12967_2017_1349_MOESM1_ESM.docx]
